# Feline morbillivirus infection associated with fatal encephalitis in a Bengal cat

**DOI:** 10.1111/jvim.16916

**Published:** 2023-10-28

**Authors:** Kara L. D. Dawson, Nicole Wildi, Mauro Cavalli, Dennis Rubbenstroth, Anna Oevermann, Torsten Seuberlich

**Affiliations:** ^1^ Division of Neurological Sciences, Vetsuisse Faculty University of Bern Bern Switzerland; ^2^ Veterinary Practice Mauro Cavalli Locarno Switzerland; ^3^ Institute for Diagnostic Virology, Friedrich‐Loeffler‐Institute, Federal Research Institute for Animal Health Isle of Riems Germany

**Keywords:** feline encephalitis, feline morbillivirus, metagenomics, neurology

## Abstract

Feline morbillivirus (FeMV) is a recently discovered morbillivirus of the family *Paramyxoviridae*, which include several highly contagious viruses with zoonotic potential. In this case report we describe the detection of FeMV in archived brain tissue of a 2‐month‐old Bengal cat with nonsuppurative encephalitis from the year 2011 in Switzerland by high‐throughput sequencing (HTS). Our metagenomics approach was able to obtain a full‐length sequence covering the entire FeMV genome. Phylogenetic analysis showed that our FeMV strain clustered within FeMV genotype 1. We were able to detect FeMV RNA by in situ hybridization (ISH) in brain sections with inflammatory lesions and demonstrated its potential neurotropism and association with encephalitis. Our results provide further insight into this recently discovered morbillivirus and encourage further investigations into the pathogenesis and epidemiology of associated diseases in cats and potentially other species.

**Abbreviations**
F: fusion glycoprotein
FeMV: feline morbillivirus
FFPE: formalin‐fixed and paraffin embedded
HTS: high‐throughput sequencing
ISH: in situ hybridization
N: nucleoprotein
P: phosphoprotein

## 1 INTRODUCTION

The genus *Morbillivirus* of the family *Paramyxoviridae* includes various viruses responsible for highly contagious diseases in humans and animals, such as measles virus, rinderpest virus and canine distemper virus.^1^ A novel morbillivirus, feline morbillivirus (FeMV), was discovered in stray cats in Hong Kong and mainland China in 2012.^2^ The FeMV genome is a single‐stranded, negative sense RNA with a genome size of approximately 16 kB.^2^ Since its discovery, it has been reported in domestic cats worldwide, mostly detected in urine samples.^3^ A causative association between FeMV infection and chronic kidney disease in cats has been proposed but remains uncertain.^3^ Recently, experimental FeMV infection in cats resulted in an acute morbillivirus‐like disease targeting immune cells at early stages and the kidney at later stages.^4^ Although in other host species several morbilliviruses show a strong neuronal tropism associated with acute and persistent encephalitis, it is unknown whether this tropism also applies to FeMV.^5^ Here, we report a case of a Bengal cat with fatal encephalitis in association with FeMV brain infection, emphasizing the neurovirulent potential of FeMV.

## 2 CASE PRESENTATION AND DISCUSSION

**FIGURE 1:**
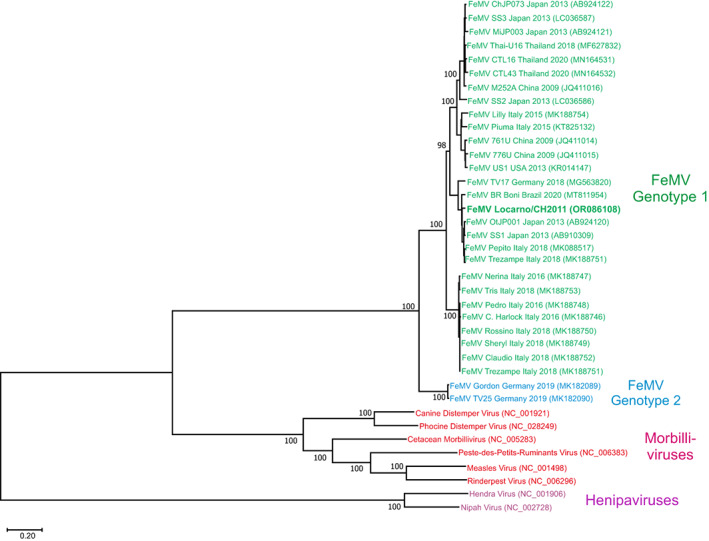
Phylogenetic comparison of whole genome sequences of representative FeMV strains and members of the genus Morbillivirus and genus Henipavirus. A phylogenetic analysis showing the clustering of the strain FeMV Locarno CH 2011 identified in this case report with FeMV strains of genotype 1. Representative full‐length sequences were obtained from NCBI GenBank with corresponding accession numbers shown in brackets. Colors indicate the genera (red, purple) and the feline morbillivirus (FeMV) genotypes (green, blue).

**FIGURE 2:**
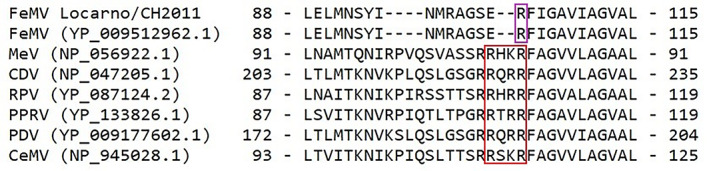
Multiple alignment of the amino acid sequence surrounding the putative cleavage site of the fusion (F) glycoprotein of representative morbilliviruses. Red: polybasic cleavage sites of morbilliviruses other than feline morbillivirus (FeMV). Purple: monobasic cleavage site with a single basic arginine (R) residue of the FeMV reference strain and the strain identified in this case report “FeMV Locarno/2011.” Corresponding protein accession numbers obtained from NCBI GenBank are shown in brackets. The start and end position of the partial amino acid sequence within the fusion protein are indicated. CDV, canine distemper virus; CeMV, cetacean morbillivirus; FeMV, feline morbillivirus; MeV, measles virus; PDV, phocine distemper virus; PPRV, peste‐des‐petits‐ruminants virus; RPV, rinderpest virus.

In 2011, a 2‐month‐old female Bengal cat was admitted to a veterinary practice in the Canton of Ticino, Switzerland. The kitten was underweight and received initial medical treatment including an antiparasitic, antibiotics and corticosteroids. In the following days however, the neurological condition of the kitten rapidly deteriorated with apathy, stupor, anisocoria and aggressive behavior. None of the siblings nor the queen was sick. Because of lack of improvement, the kitten was euthanized, and the brain was removed and submitted to the Division of Neurological Sciences of the Vetsuisse Faculty of Bern for further neuropathological investigations. Histopathology identified an unusually mild lymphocytic inflammation of the brain associated with neuronal degeneration and necrosis consistent with an acute viral infection. Lesions were most prominent in the thalamus, basal ganglia, hippocampus, and brainstem. We also observed inclusion bodies in the soma of neurons. We performed further etiologic investigations by immunohistochemistry (rabies virus, canine distemper virus, tick‐borne encephalitis virus [in 2011]) as well as reverse transcription PCR (rustrela virus and Borna disease virus 1 [in 2022]) and all were negative (Supplemental Information). We extracted total RNA from formalin‐fixed and paraffin embedded (FFPE) brain material, prepared a cDNA library and sequenced it at a read depth of approximately 130 M reads with 100 cycles in single‐end mode (Supplemental Information). We analyzed the sequencing data using an in‐house virus discovery bioinformatics pipeline (Supplemental Information) and found a viral hit for FeMV. By de novo assembly, we generated a contiguous sequence of 16,050 nucleotides (nt; read depth 347×) corresponding to a full FeMV genome. A nucleotide BLAST search identified the highest similarity with the FeMV strain “SS1” isolated from domestic cats in Japan in 2014, with an overall high nucleotide identity of 97.1%.^6^ Pairwise identity on amino acid level ranged from 98.2% to 99.4% (Table S2). We designated this strain as FeMV Locarno/CH2011 (GenBank accession no. OR086108). Typical for morbilliviruses, the FeMV Locarno/CH2011 genome fulfills the “rule of 6” (ie, it is a multiple of 6 nt). It is comprised of 6 nonoverlapping genes (3′‐N, P/V/C, M, F, H, L‐5′) that encode for 6 structural proteins and 2 additional nonstructural proteins overlapping the phosphoprotein (P).^1^ Phylogenetic analysis showed clustering of FeMV Locarno/CH2011 with FeMV strains of genotype 1 (Figures 1 and S1). In contrast to other morbilliviruses, but in agreement with previously reported FeMV, the fusion (F) glycoprotein lacks a polybasic furin cleavage signal but has a single basic residue at the putative cleavage site within the F protein (Figure 2).^4^ Instead of furin, cathepsin has been found to cleave the FeMV F protein, a feature that is characteristic of the closely related zoonotic and neuropathogenic henipaviruses.^4^

**FIGURE 3:**
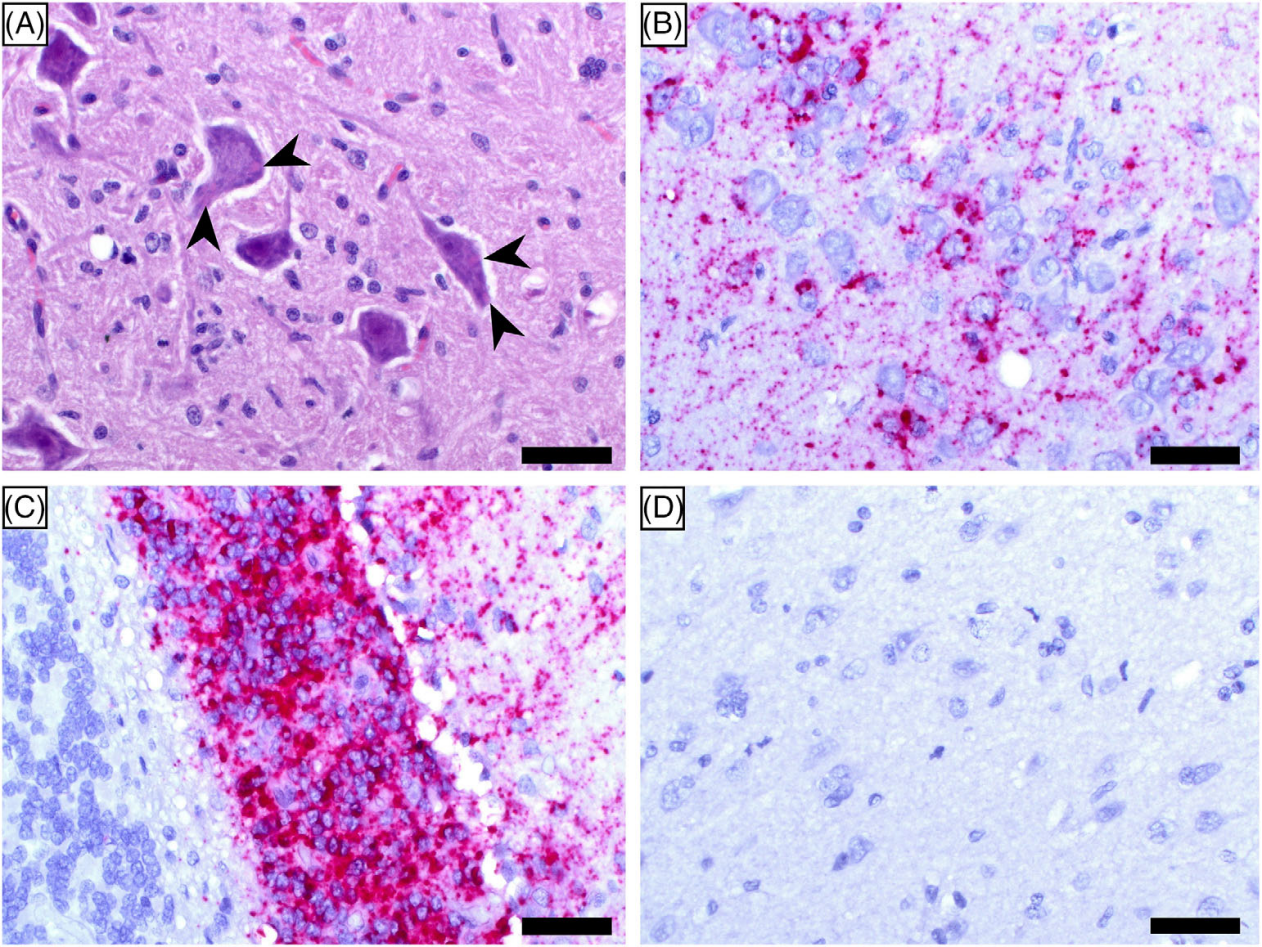
Histopathological findings and detection of feline morbillivirus (FeMV) RNA in the brain by in situ hybridization (ISH). (A) Hematoxylin and eosin (H&E) staining of the brainstem showing neurons with one to multiple eosinophilic intracytoplasmic inclusion bodies of various size (arrowheads). (B) In situ detection of FeMV RNA shown as red granular staining located in pyramidal cells of the hippocampus. (C) Positive signals for FeMV RNA in the granular cell layer of the cerebellum. (D) Negative control of a cat brain that did not have histopathological lesions without in situ detection of FeMV RNA. Scale bars: 20 μm.

We performed in situ hybridization (ISH) using a probe targeting the nucleoprotein (N) gene of FeMV and detected an abundance of FeMV RNA in the soma of neurons and astrocytes (Figure 3). Notably, we observed strong ISH signals despite mild inflammation. The inadequate immune response might have been an effect of the immunosuppressive properties of FeMV and could have been further accentuated by medical treatment with corticosteroids. Although there have been no reports on FeMV associated with encephalitis in cats so far, investigations of the cellular tropism of FeMV indicated its potential to infect primary brain cells of cats in vitro.^7^ Overall, our data provide plausible evidence for a causative association between FeMV infection and encephalitis in cats.

## 3 CONCLUSION

Morbilliviruses are a major threat to human and animal health and some have a high potential for cross species transmission.^1^ Our results contribute to a better awareness of FeMV as a viral and presumptive neurotropic pathogen in cats. It is important to increase our understanding of the associated host and pathogen factors that are involved in FeMV pathogenesis and the epidemiology of associated diseases in cats and potentially other species.

## CONFLICT OF INTEREST DECLARATION

Authors declare no conflict of interest.

## OFF‐LABEL ANTIMICROBIAL DECLARATION

Authors declare no off‐label use of antimicrobials.

## INSTITUTIONAL ANIMAL CARE AND USE COMMITTEE (IACUC) OR OTHER APPROVAL DECLARATION

Authors declare no IACUC approval was needed. Owners of the animals of which tissue samples were used retrospectively in this study provided informed consent for the use of these materials for research purposes.

## HUMAN ETHICS APPROVAL DECLARATION

Authors declare human ethics approval was not needed for this study.

## Supporting information


**Data S1:** Supporting Information.

**Figure S1:** Phylogenetic analysis of FeMV strains based on a partial nucleotide segment of the large polymerase protein (L). Representative FeMV strains were obtained from NCBI GenBank with corresponding accession numbers shown in brackets. Colors indicate the genotypes (green, blue) and clades (orange, pink) of FeMV strains.

**Table S1:** Primers and probes used for orthobornavirus and rustrela virus (RusV) RNA detection.

**Table S2:** Whole genome comparison between the discovered feline morbillivirus (FeMV) strain Locarno/CH2011 and the FeMV reference strain and FeMV strain “SS1.”
