# Supplementary material for: Feline morbillivirus infection associated with fatal encephalitis in a Bengal cat
Source: J Vet Intern Med. 2023 Oct 28;37(6):2510–3. doi: 10.1111/jvim.16916 (PMC10658552; doi:10.1111/jvim.16916)
Supplement: Supplementary file 1 — Data S1: Supporting Information. [file JVIM-37-2510-s002.pdf]

Supplemental information

Tissue sample

We obtained the case of the two-month-old Bengal cat diagnosed with a non-suppurative encephalitis suggestive of a viral infection from the archive of the Division of Neurological Sciences, Vetsuisse Faculty, University of Bern, Switzerland. We reviewed the documented patient history and evaluated histology slides of different brain regions for typical hallmarks of a viral encephalitis, which includes perivascular cuffs of mononuclear cells, reactive gliosis and neuronal necrosis.[1]

Sample preparation and high-throughput sequencing (HTS)

From formalin-fixed paraffin embedded (FFPE) brain samples, we cut four 10 µm sections for total RNA extraction using the RNeasy FFPE kit (QIAGEN) after deparaffinization with xylol. The RNA concentration was assessed with the Qubit™ 4 Fluorometer (Invitrogen) and quantified with the 5200 Fragment Analyzer System (Agilent). We prepared a cDNA library using the CORALL Total RNA-Seq Library kit (Lexogen), which was sequenced in an Illumina NovaSeq6000 machine in single-end mode for 100 cycles at a read depth of ~130 Mio. reads. Raw reads are available at the National Center for Biotechnical Information (NCBI) sequence read archive (accession no. PRJNA980262).

Bioinformatics Analysis

We analyzed HTS reads using an in-house established bioinformatics pipeline.[2] A total of 137'750'925 raw reads were subjected to a quality control and trimming process using fastqc (Ver. 0.11.7)[3] and fastp (Ver. 0.12.5)[4], respectively. After removal of reads that mapped to the cat reference genome (Felis catus Fca126) using STAR (Ver. 2.7.3a)[5], the

remaining 21'226'267 unmapped and quality selected reads were mapped to the RefSeq viral genome database using bowtie2 (Ver. 2.3.4.1)[6]. The resulting 244'306 reads mapped to the feline morbillivirus reference genome (FeMV, NCBI GenBank accession number NC_039196.1) with a coverage of 83.46% and a mean read depth of 1188.76. To obtain longer contiguous sequences (contigs) > 500 nt, we *de novo* assembled reads using SPAdes (Ver. 3.12.0)[7]. We subsequently analyzed the detected FeMV contig using the Geneious Prime 2023.1 software package (https://www.geneious.com).

RT-qPCR

For the detection of RNA of Borna disease virus 1 (BoDV-1), two different TaqMan RT-qPCR assays, BoDV-1 Mix-1 and Mix-6, were employed. In addition, the assay panBorna v7.2 was applied for the detection of RNA of a broad range of mammalian and avian orthobornaviruses.[8] All assays were performed as described previously.[9] Primers and probes are listed in Table S1. RNA of rustrela virus (RusV) was detected by TaqMan RT-qPCR assay panRusV-2 as described previously by Matiasek et al (Table S1).[10]

A BRYT green assay was used for the detection of FeMV with the GoTaq One-Step RT-qPCR (Promega) kit. We *in silico* assembled FeMV consensus primers (Forward 3'-CCCTAGGGCAGCACATGAAA-5', Reverse 3'-GCTAGCACGAATCAACCCCT-5'), targeting a 100 nt fragment of the conserved large polymerase (L) gene of morbilliviruses.[11] The assay ran on a 7300 Real Time PCR system (Applied Biosystems, Singapore) with the following conditions: 40°C for 15 min, 95°C for 10 min, 40 cycles of 95°C for 10 s, 56°C for 30 s, 72°C for 30s. RNase-free water was used a negative control. A cut-off value for the detection of FeMV was set at a cq value of 35.

In situ hybridization

Detection of FeMV RNA by *in situ* hybridization (ISH) was performed on FFPE cat brain tissue sections of 3 µm thickness. An ISH probe (V-FeMV-N-sense-C1) targeting the positive strand of the N protein of FeMV (nucleotide position 278-1395 of MT811954.1: cat no. 119819) was ordered from Advanced Cell Diagnostics. ISH was performed with the RNAscope® 2.5 HD Reagent Kit-RED (Advanced Cell Diagnostics) according to the manufacturer's instructions.

Phylogenetic analysis

A phylogenetic tree was constructed based on the full genome sequence of the obtained strain FeMV Locarno/CH2011 (Figure 1). We obtained whole genomes of representative FeMV strains as well as members of the genus *Morbillivirus* and genus *Henipavirus* within the family *Paramyxoviridae* from NCBI GenBank. We also constructed a phylogenetic tree based on a partial segment (370 nt) of the large polymerase protein (L) from available FeMV strains (Figure S1). All sequences were aligned with the MAFFT (Ver. 7.475)[12] software. A maximum-likelihood phylogenetic analysis was performed with IQ-Tree (Ver. 2.0.3)[13] with 1000 bootstrap replicates.

Sequence comparisons

The putative cleavage site of the fusion (F) glycoprotein of morbilliviruses was compared using Geneious Prime. We aligned a partial sequence of the F protein containing the monobasic cleavage site of FeMV and the polybasic cleavage site of other morbilliviruses, respectively (Figure 2). We further compared the full-length sequence of FeMV Locarno/CH2011 to the reference strain FeMV '761U' and the strain FeMV 'SS1' with the highest pairwise identity based on a BLASTn search. Full-length sequences were aligned

using Geneious Prime, resulting in pairwise identities on nucleotide and amino acid level (Table S2).

Supplemental References

1. Vandevelde M, Higgins R, Oevermann A. *Veterinary Neuropathology*. John Wiley & Sons, Chichester, United Kingdom, 2012: 49-50
2. Kauer RV, Koch MC, Hierweger MM, et al. Discovery of novel astrovirus genotype species in small ruminants. *PeerJ* 2019;7:e7338
3. Andrews, S. FastQC: a quality control tool for high throughput sequence data. 2010. Available from: http://www.bioinformatics.brabraham.ac.uk/projects/fastqc/ (accessed August 2023)
4. Chen S, Zhou Y, Chen Y, et al. fastp: an ultra-fast all-in-one FASTQ preprocessor. *Bioinformatics* 2018;34(17):i884-i890
5. Dobin A, Davis CA, Schlesinger F, et al. STAR: ultrafast universal RNA-seq aligner. *Bioinformatics* 2013;29(1):15-21
6. Langmead B, Salzberg SL. Fast gapped-read alignment with Bowtie 2. *Nat Methods* 2012;9(4):357-9
7. Nurk S, Bankevich A, Antipov D, et al. Assembling single-cell genomes and mini-metagenomes from chimeric MDA products. *J Comput Biol* 2013;20(10):714-37
8. Sigrist B, Geers J, Albini S, et al. A New Multiplex Real-Time RT-PCR for Simultaneous Detection and Differentiation of Avian Bornaviruses. *Viruses* 2021;13(7):1358
9. Schlottau K, Forth L, Angstwurm K, et al. Fatal Encephalitic Borna Disease Virus 1 in Solid-Organ Transplant Recipients. *N Engl J Med* 2018;379(14):1377-1379

10. Matiasek K, Pfaff F, Weissenböck H, et al. Mystery of fatal 'staggering disease' unravelled: novel rustrela virus causes severe meningoencephalomyelitis in domestic cats. *Nat Commun* 2023;14(1):624

11. Woo PC, Lau SK, Wong BH, et al. Feline morbillivirus, a previously undescribed paramyxovirus associated with tubulointerstitial nephritis in domestic cats. *Proc Natl Acad Sci U S A* 2012;109(14):5435-5440

12. Katoh K, Standley DM. MAFFT multiple sequence alignment software version 7: improvements in performance and usability. *Mol Biol Evol* 2013;30(4):772-80

13. Minh BQ, Schmidt HA, Chernomor O, et al. IQ-TREE 2: New Models and Efficient Methods for Phylogenetic Inference in the Genomic Era. *Mol Biol Evol* 2020;37(5):1530-1534
