# Supplementary material for: Feline morbillivirus infection associated with fatal encephalitis in a Bengal cat
Source: J Vet Intern Med. 2023 Oct 28;37(6):2510–3. doi: 10.1111/jvim.16916 (PMC10658552; doi:10.1111/jvim.16916)
Supplement: Supplementary file 3 — Table S1: Primers and probes used for orthobornavirus and rustrela virus (RusV) RNA detection. [file JVIM-37-2510-s004.pdf]

**Table S1: Primers and probes used for orthobornavirus and rustrela virus (RusV) RNA detection.**

| Assay | Primer/Probe name | Sequence (5' to 3') | Suppl. Ref. |
|---|---|---|---|
| panBorna v7.2 | Borna-1319-F | CGCGACCMTCGAGYCTRGT | (*9*) |
| | Borna-1471.2-FAM | FAM-AAGAAYCCHTCCATGATCTCMGAYCMAGA-BHQ1 | |
| | Borna-1529-R | GACARCTGYTCCCTTCCKGT | |
| BoDV-1 Mix-1 | BoDV-1_1258+ | TAGTYAGGAGGCTCAATGGCA | (*9*) |
| | BoDV-1_1316_FAM | FAM-AAGAAGATCCCCAGACACTACGACG-BHQ1 | |
| | BoDV-1_1419- | GTCCYTCAGGAGCTGGTC | |
| panRusV-2 | RusV_234+ | CCCCGTGTTCCTAGGCAC | (*10*) |
| | RusV_256_P | FAM-GTGAGCGACCACCCAGCACTCCA-BHQ1 | |
| | RusV_323- | TCGCCCCATTCWACCCAATT | |
