# Supplementary material for: Feline morbillivirus infection associated with fatal encephalitis in a Bengal cat
Source: J Vet Intern Med. 2023 Oct 28;37(6):2510–3. doi: 10.1111/jvim.16916 (PMC10658552; doi:10.1111/jvim.16916)
Supplement: Supplementary file 4 — Table S2: Whole genome comparison between the discovered feline morbillivirus (FeMV) strain Locarno/CH2011 and the FeMV reference strain and FeMV strain “SS1.” [file JVIM-37-2510-s001.pdf]

Table S2: Whole genome comparison between the discovered feline morbillivirus (FeMV) strain Locarno/CH2011 and the FeMV reference strain and FeMV strain 'SS1'.

Genomic region	Nucleotide position	FeMV NC_039196.1 Pairwise identity		FeMV strain SS1 Pairwise identity	
		[nt]	[aa]	[nt]	[aa]
**3' untranslated region**	1 - 107	96.1%	n.a.	97.1%	n.a.
**Nucleocapsid protein**	108 - 1667	90.1%	96.1%	97.3%	98.7%
**Intergenic region**	1668 - 1780	82.3%	n.a.	93.8%	n.a.
**Phosphoprotein**	1781 - 3256	88.9%	86.8%	97.5%	95.5%
**Intergenic region**	3257 - 3388	79.5%	n.a.	97.7%	n.a.
**Matrix protein**	3389 - 4402	89.7%	95.3%	97.4%	99.4%
**Intergenic region**	4403 - 4953	70.6%	n.a.	93.5%	n.a.
**Fusion protein**	4954 - 6585	89.3%	94.0%	97.4%	98.9%
**Intergenic region**	6586 - 6962	75.6%	n.a.	94.2%	n.a.
**Hemagglutinin protein**	6963 - 8750	90.2%	94.8%	96.8%	98.2%
**Intergenic region**	8751 - 8891	71.6%	n.a.	95.7%	n.a.
**Large polymerase protein**	8892 - 15500	89.8%	96.0%	97.9%	99.3%
**5' untranslated region**	15501 - 16050	72.5%	n.a.	91.9%	n.a.

FeMV: Feline morbillivirus. nt: nucleotide. aa: amino acid. n.a.: not applicable.
