# Supplementary material for: Feline morbillivirus infection associated with fatal encephalitis in a Bengal cat
Source: J Vet Intern Med. 2023 Oct 28;37(6):2510–3. doi: 10.1111/jvim.16916 (PMC10658552; doi:10.1111/jvim.16916)
Supplement: Supplementary file 2 — Figure S1: Phylogenetic analysis of FeMV strains based on a partial nucleotide segment of the large polymerase protein (L). Representative FeMV strains were obtained from NCBI GenBank with corresponding accession numbers shown in brackets. Colors indicate the genotypes (green, blue) and clades (orange, pink) of FeMV strains. [file JVIM-37-2510-s003.pdf]

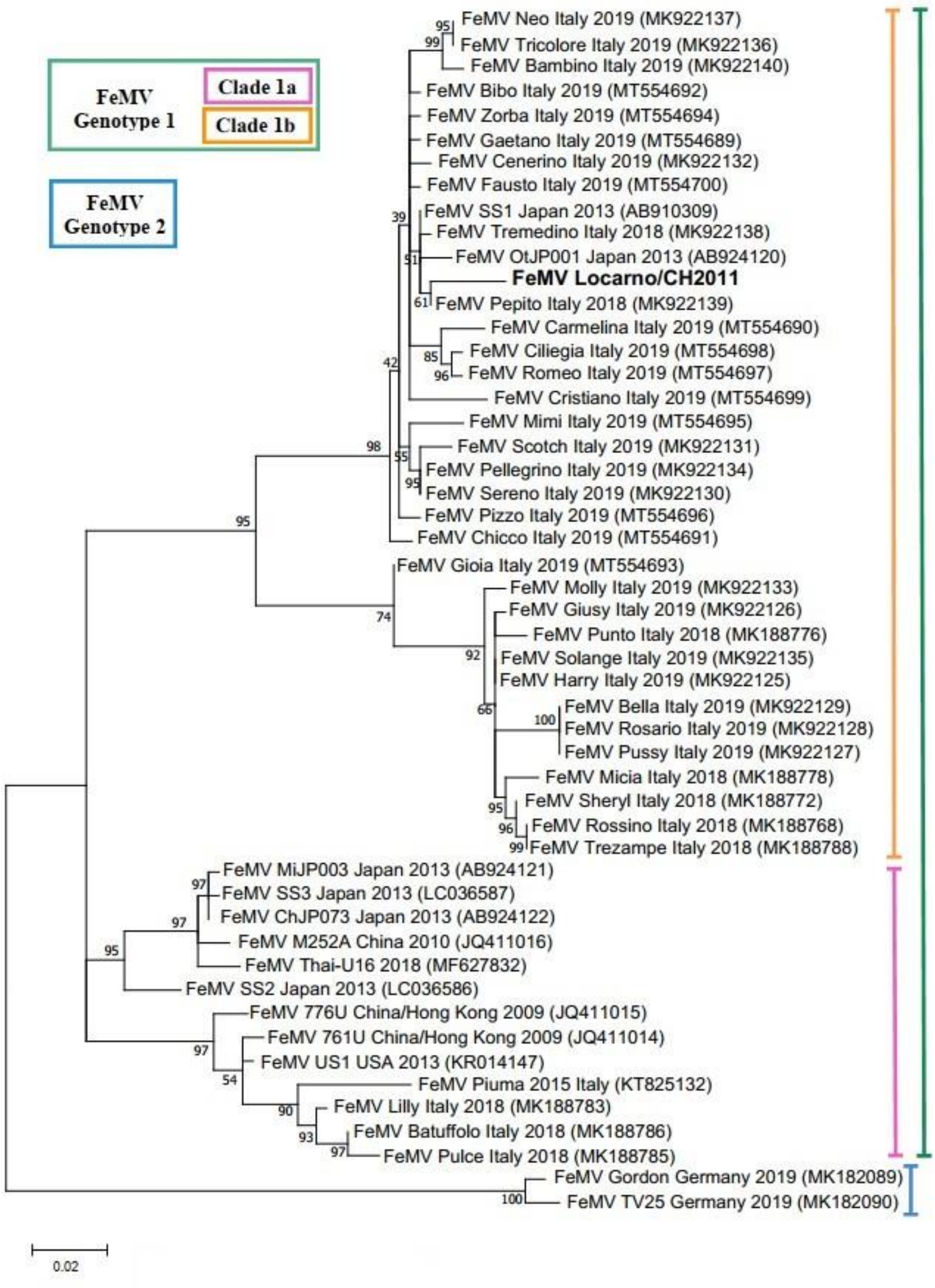

Supplementary Figure S1: Phylogenetic analysis of FeMV strains based on a partial nucleotide segment of the large polymerase protein (L). Representative FeMV strains were obtained from NCBI GenBank with corresponding accession numbers shown in brackets. Colors indicate the genotypes (green, blue) and clades (orange, pink) of FeMV strains.
